# A Cross-Sectional Analysis of Bystander Cardiopulmonary Resuscitation (CPR): Behavioural Changes in the COVID-19 Era

**DOI:** 10.7759/cureus.62299

**Published:** 2024-06-13

**Authors:** John Shadarevian, Jim Li, Katherine S Allan, Brian Grunau, John Sapp, Santokh Dhillon, Sumeet S Saini, Adrija Chakrabarti, Santabhanu Chakrabarti

**Affiliations:** 1 Department of Medicine, Dalhousie University, Halifax, CAN; 2 Department of Surgery, Division of Radiation Oncology, University of British Columbia, Vancouver, CAN; 3 Division of Cardiology, St. Michael's Hospital, Toronto, CAN; 4 Department of Medicine, University of British Columbia, Vancouver, CAN; 5 Division of Cardiology, Izaak Walton Killam (IWK) Children's Heart Centre, Halifax, CAN

**Keywords:** pandemic response, personal protective equipment, out of hospital cardiac arrest, covid-19, bystander cpr

## Abstract

Objectives

The coronavirus disease 2019 (COVID-19) pandemic has impacted public health systems and individuals' behaviour, with decreasing survival rates among out-of-hospital cardiac arrest (OHCA) patients. Bystander cardiopulmonary resuscitation (CPR) improves OHCA outcomes, which may have been affected by COVID-19. We sought to understand the impacts of COVID-19 on bystanders' willingness to administer CPR in three Canadian provinces.

Methods

Participants ≥ 18 years of age were surveyed online about their current and recalled pre-pandemic attitudes toward CPR and perceived transmission risk. We compared mean willingness to perform various CPR actions before and during the pandemic using paired t-tests. Differences in willingness across three provinces were assessed using analysis of variance (ANOVA) and Tukey's Honestly Significant Difference (HSD) test. We also conducted Chi-square tests to assess changes in willingness to perform CPR on children and older adults.

Results

Five hundred thirty-five participants were surveyed from October 1 to November 15, 2021. The mean age was 42.7 years (SD 14.5), and 60.2% were female. Participants reported less willingness to perform chest compressions on strangers during the pandemic compared to their recollections before the pandemic (mean willingness 86.2% vs. 94.3% prior, p<0.001). With personal protective equipment (PPE) available, particularly masks, willingness recovered to 91.3% (p<0.001). Willingness was higher in Nova Scotia (NS) than in British Columbia (BC) or Ontario (ON). Reluctance to assist older adults increased from 6.6% to 12.0% (p=0.020).

Conclusions

This study highlights changes in CPR willingness during the COVID-19 pandemic, underscoring the importance of PPE and offering insights into public health strategies pertaining to CPR during a pandemic.

## Introduction

The global health crisis induced by coronavirus disease 2019 (COVID-19) has profoundly impacted the international community, public health systems, and economies [1-3]. Besides the impact on those infected, the pandemic has also had many indirect public health consequences, namely disruptions to routine care and health-seeking behaviours [4-6]. A decrease in survival for patients experiencing out-of-hospital cardiac arrest (OHCA) was observed [7,8]. Several factors may underlie this, including lengthier emergency medical service response times, diminished healthcare access, and reluctance among bystanders to initiate cardiopulmonary resuscitation (CPR) [9,10].

Cardiac arrest remains a prominent contributor to global mortality rates [11]. Bystander-administered CPR is fundamental in improving survival probabilities and neurological outcomes [12,13]. However, the COVID-19 pandemic may have instilled a reluctance to perform CPR due to apprehension about contracting COVID-19. Factors influencing willingness to perform CPR may include the relationship between the patient and the bystander, the availability of personal protective equipment (PPE), CPR training, and COVID-19 vaccination. Prior research examining the pandemic’s impact revealed a decline in bystander-initiated CPR despite increases in OHCA [7,8,14]. An Italian study during the pandemic's early phase reported a reduction in bystander CPR rates from 31% to 20%. A study in France observed a decline in the frequency of bystander CPR from 63.9% to 47.8% throughout the pandemic. Racial disparities and socioeconomic factors, well-recognized barriers to bystander CPR, may be further exacerbated during the pandemic. Marginalized and underserved populations, often burdened with higher rates of cardiac arrest, may have been significantly impacted due to the pandemic-induced CPR reluctance, deepening the divide in OHCA outcomes [15,16].

Despite declining bystander CPR during the COVID-19 pandemic, research is lacking on the elements influencing a bystander's decision to perform CPR. Understanding these determinants is essential for formulating public health strategies and CPR instruction programs to confront impediments to CPR. Moreover, this understanding is crucial to addressing deepening racial and socioeconomic disparities in OHCA outcomes during public health crises. We sought to assess factors that impacted bystanders' willingness to perform CPR in the context of the COVID-19 pandemic.

## Materials and methods

Study design

We conducted a cross-sectional study at the University of British Columbia, the University of Toronto, and Dalhousie University, employing a convenience sampling method to recruit participants from the public through digital mediums such as Facebook, Twitter, and Instagram. The accounts of the Hearts of British Columbia (BC) Foundation and the investigators were used. Eligibility for participation required individuals to be at least 18 years old and able to comprehend and read English. Incentives were not provided. They were informed that our study explored how the COVID-19 pandemic influenced bystanders' willingness to perform CPR in different situations.

Survey instrument

We developed our survey using Qualtrics, a cloud-based platform (www.qualtrics.com), adhering to the British Columbia Freedom of Information and Protection of Privacy Act (FIPPA). The questionnaire covered participants' demographics (including age, gender, education level, and occupation), COVID-19-related experiences, experience with CPR, and willingness to perform CPR in various scenarios depending on the relationship between rescuer and patient and PPE availability. For comparison, we asked participants to report their perceived willingness to perform multiple CPR actions before and during the pandemic. Willingness was measured continuously from 0% (no willingness) to 100% (complete willingness). The survey took about eight minutes to finish and did not gather any personally identifiable information. We pilot-tested the questionnaire with a small group of participants to ensure clarity, readability, and face validity.

Ethical considerations

The Research Ethics Boards of the University of British Columbia, the University of Toronto (approval number 21-83496), and Dalhousie University (approval number 20213729) granted ethics approval for this study. We obtained informed consent at the start of the survey, informing the participants of the study's purpose, the lack of known risks associated with participating, their voluntary participation, and that they could refuse to participate or withdraw at any time.

Data collection and analysis

We collected survey data over six weeks, from October 1 to November 15, 2021. We described participants' demographics and attitudes toward CPR using mean with 95% confidence intervals (CI) for continuous variables and counts with percentages for categorical variables.

We first employed paired t-tests to compare the mean percentage of willingness to perform various CPR actions before and during the pandemic. Following this, we compared responses by the three provinces with the highest number of participants: Ontario (ON), British Columbia (BC), and Nova Scotia (NS). Differences in the mean percentage of willingness to perform various CPR actions on a stranger during the pandemic were assessed using analysis of variance (ANOVA). In cases where ANOVA revealed a significant difference, Tukey's Honestly Significant Difference (HSD) test was employed for pairwise comparisons.

Subsequently, we performed additional paired t-tests to compare the mean percentage of willingness to perform chest compressions during the pandemic under various scenarios (without and with personal protective equipment, flu-like symptoms, COVID-19 risk information, and close relations who had tested positive). To assess the influence of the pandemic on participants' willingness to perform CPR on children and older adults, age 65 years and older, we conducted McNemar's test comparing the number of participants who were more, equally, or less likely to perform CPR before and during the pandemic. Statistical significance was set at p<0.05. We conducted all analyses using RStudio 4.3.0 (www.r-project.org).

Due to partial survey responses, the number of respondents for individual questions varied. We used the number of responses available for each question during statistical analysis. This approach allowed us to utilize all available data while acknowledging the potential impact of varying response rates on our findings.

## Results

Sample demographics

535 participants started the survey, but only 382 completed it in its entirety. Due to incomplete responses, the number of respondents for individual questions varied, ranging from 382 to 535. Our statistical analyses accounted for this variability in response rates, where we used the available responses for each question. 142 participants were from ON, 123 from BC, and 79 from NS. The mean age was 42.7 ± 14.5 years. 237 (60.2%) participants were female, 313 (82.0%) held a bachelor's degree or higher level of education, and 350 (88.9%) were involved in the healthcare field or had received prior CPR training. 26 (6.7%) of participants had previously tested positive for COVID-19, 145 (35.2%) had family or friends who had tested positive, and 383 (99.0%) had received at least one dose of the COVID-19 vaccine (Table 1).

**Table 1:** Demographic characteristics of survey participants COVID-19: Coronavirus disease 2019, CPR: Cardiopulmonary resuscitation

Parameter	Overall (N = 535)
Gender	
Female, n (%)	237 (60.2)
Male, n (%)	152 (38.6)
Non-binary, n (%)	3 (0.7)
Not disclosed, n (%)	2 (0.5)
Mean age (years)	42.7 ± 14.5
Country of residence	
Canada, n (%)	373 (94.9)
Ontario, n (%)	142 (36.1)
British Columbia, n (%)	123 (31.3)
Nova Scotia, n (%)	79 (20.1)
Other, n (%)	29 (7.4)
United States, n (%)	13 (3.3)
Other, n (%)	7 (1.8)
Highest education completed	
High school diploma, n (%)	38 (9.9)
Associate degree or college diploma, n (%)	31 (8.1)
Bachelor’s degree, n (%)	171 (44.8)
Master’s or professional degree, n (%)	142 (37.2)
Medical training	
None, n (%)	44 (11.2)
First aid or CPR course only, n (%)	141 (35.8)
Healthcare worker in training, n (%)	20 (5.1)
Healthcare worker, n (%)	189 (48.0)
Healthcare field	
Nursing, n (%)	70 (33.5)
Medicine, n (%)	72 (34.4)
Paramedic, n (%)	46 (22.0)
Other, n (%)	21 (10.0)
Past COVID-19 testing	
Positive, n (%)	26 (6.7)
Negative, n (%)	264 (68.4)
Never tested, n (%)	96 (24.9)
Past COVID-19 testing of family or friends	
Positive, n (%)	145 (35.2)
Negative, n (%)	209 (50.7)
Never tested, n (%)	40 (9.7)
Unaware, n (%)	18 (4.4)
COVID-19 vaccination	
1 or more doses, n (%)	383 (99.0)
No doses, n (%)	4 (1.0)

Willingness to perform CPR and influencing factors

**Figure 1:**
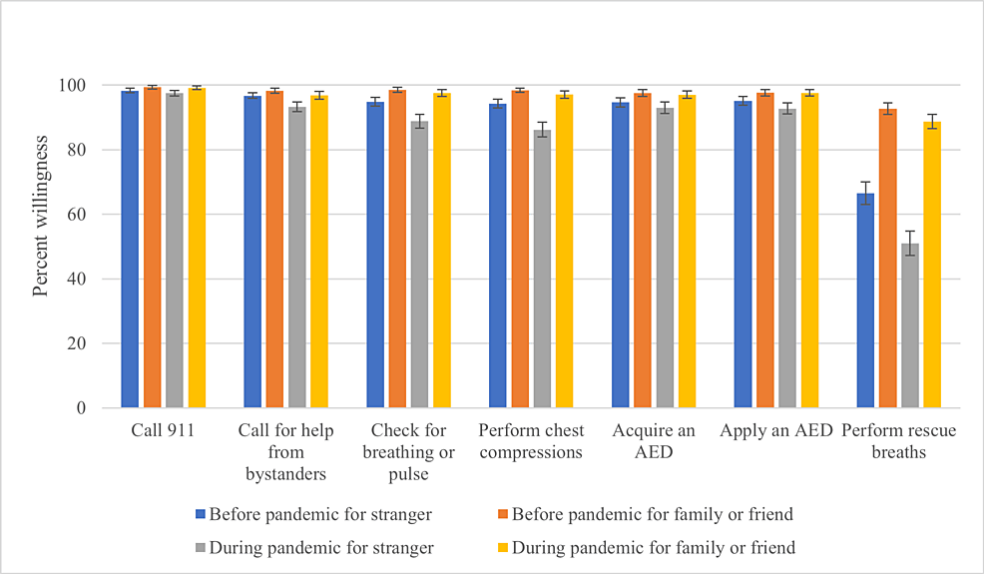
Impact of the COVID-19 pandemic on bystander willingness to perform CPR interventions Participants were asked to report their willingness to perform each CPR action before and during the COVID-19 pandemic, depending on the bystander’s relationship with the victim. Each bar represents the mean percent willingness, with error bars representing 95% confidence intervals. AED: Automated external defibrillator, CPR: Cardiopulmonary resuscitation, COVID-19: Coronavirus disease 2019

Participants expressed over 90% willingness to activate emergency medical services by calling 911, soliciting assistance from bystanders, and acquiring an automated external defibrillator (AED). However, for checking for breathing or pulse and performing chest compressions, willingness decreased to 88.9% ± 2.1 (p<0.001) and 86.2% ± 2.3 (p<0.001) during the pandemic, respectively. Healthcare providers, defined as those working or training in a healthcare field, had slightly less willingness to perform chest compressions (85.9% ± 1.7) than non-providers (88.2% ± 1.7), though the difference was not statistically significant (p=0.345). The most substantial decline was in willingness to perform rescue breaths on a stranger, falling from 66.5% ± 3.5 before the pandemic to 51.0% ± 3.8 during the pandemic (p<0.001) (Figure 1).

**Figure 2:**
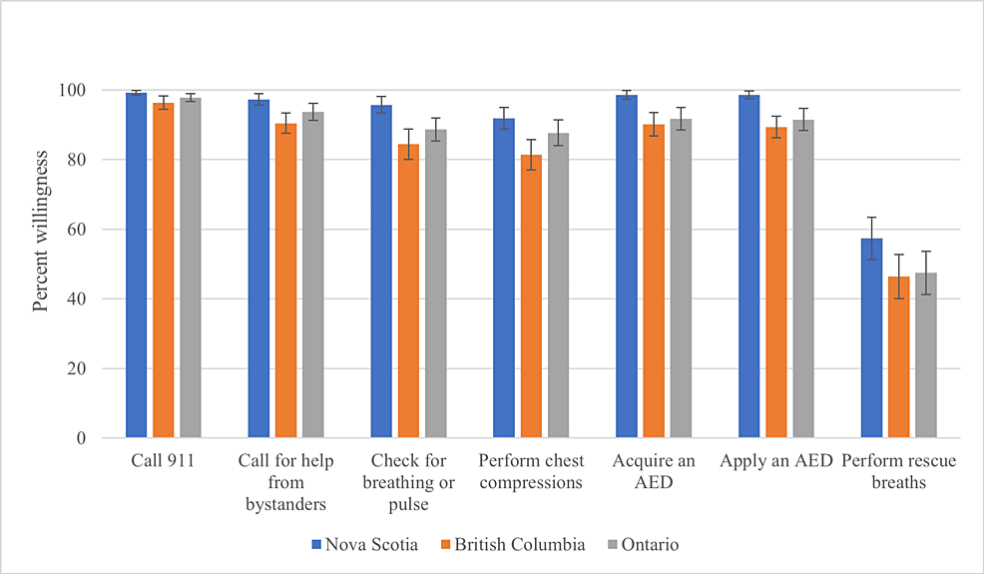
Regional variation in bystander willingness to perform CPR interventions Willingness to assist a stranger during the pandemic was determined. The mean percent willingness for the subgroups in Nova Scotia, British Columbia, and Ontario are displayed, with error bars representing 95% confidence intervals. AED: Automated external defibrillator, CPR: Cardiopulmonary resuscitation

Participants from NS reported significantly higher willingness to call for help from bystanders (97.3% ± 2.2) than BC (90.4% ± 3.1, p=0.003) and a greater willingness to check for breathing or pulse (95.7% ± 3.1) than BC (84.5% ± 4.7, p<0.001) and ON (88.7% ± 3.3, p=0.042). There was higher readiness to perform chest compressions in NS (91.8% ± 4.2) relative to BC (81.4% ± 4.7, p=0.003). Participants from NS were more likely to both acquire (98.6% ± 1.7 vs. BC 90.2% ± 3.6, p=0.002 and ON 91.7% ± 3.2, p=0.015) and apply an AED (98.6% ± 1.5 vs. BC 89.4% ± 3.3, p<0.001 and ON 91.5% ± 3.2, p=0.007). We did not detect differences between provinces when examining willingness to call 911 or perform rescue breaths (Table 2, Figure 2).

**Table 2:** Comparison of bystander willingness to perform CPR interventions by province. ANOVA: Analysis of variance, AED: Automated external defibrillator, BC: British Columbia, CPR: Cardiopulmonary resuscitation, HSD: Honestly significant difference, NS: Nova Scotia, ON: Ontario

CPR Intervention	Mean percent willingness (± 95% CI)			ANOVA p-value	Tukey’s HSD p-value		
	NS (N = 79)	BC (N = 123)	ON (N = 142)		NS vs. BC	NS vs. ON	BC vs. ON
Call 911	99.2 ± 0.8	96.4 ± 2.0	97.8 ± 1.1	0.057	-		
Call for help from bystanders	97.3 ± 2.2	90.4 ± 3.1	93.7 ± 2.4	0.007	0.003	0.182	0.253
Check for breathing or pulse	95.7 ± 3.1	84.5 ± 4.7	88.7 ± 3.3	0.001	<0.001	0.042	0.324
Perform chest compressions	91.8 ± 4.2	81.4 ± 4.7	87.7 ± 3.7	0.006	0.003	0.390	0.118
Acquire an AED	98.6 ± 1.7	90.2 ± 3.6	91.7 ± 3.2	0.004	0.002	0.015	0.797
Apply an AED	98.6 ± 1.5	89.4 ± 3.3	91.5 ± 3.2	0.001	<0.001	0.007	0.639
Perform rescue breaths	57.3 ± 8.1	46.4 ± 6.8	47.5 ± 6.2	0.102	-		

**Figure 3:**
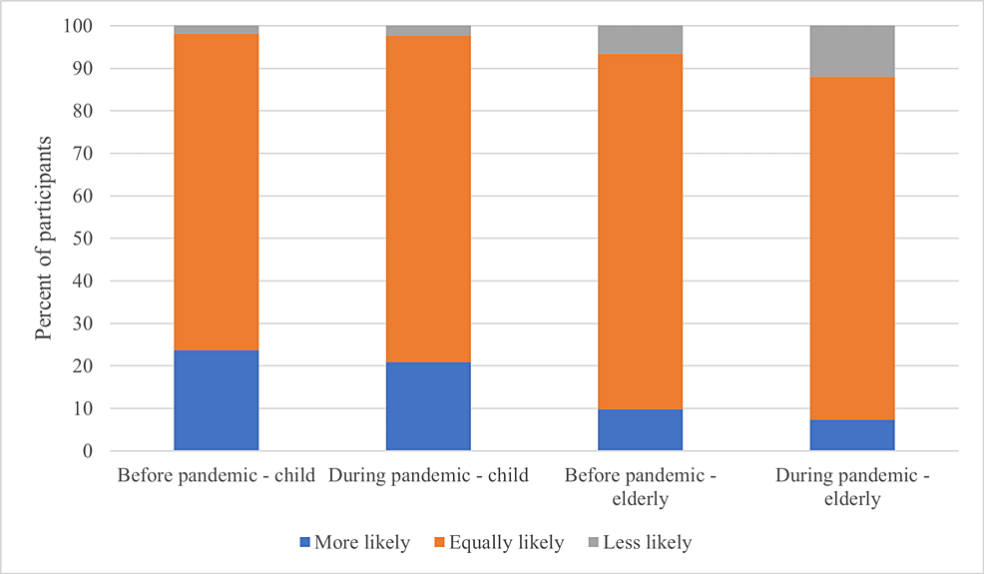
Willingness to perform CPR on children and elderly individuals Participants were asked how likely they were to perform CPR on children and the elderly, age 65 years and older, compared with adults before and during the COVID-19 pandemic. CPR: Cardiopulmonary resuscitation, COVID-19: Coronavirus disease 2019

When asked to recall their attitudes before and during the pandemic, the number of participants who were more willing to perform CPR on children dropped from 94 (23.7%) before the pandemic to 82 (20.9%) during the pandemic (p<0.001). On the other hand, the number of participants less inclined to perform CPR for the elderly, age 65 years and older, increased significantly from 26 (6.6%) before the pandemic to 47 (12.0%) during the pandemic (p<0.001) (Figure 3).

PPE availability and flu-like symptoms in the rescuer were associated with changes in the willingness to perform chest compressions. We observed that 180 (41.1%) participants were more willing to perform chest compressions when gloves were available, and 281 (64.2%) were more willing to perform chest compressions when a mask was available for the rescuer or the victim. During the pandemic, the willingness to perform chest compressions on a stranger decreased from 94.3% ± 1.4 to 86.2% ± 2.3 (p<0.001) and even further to 74.8% ± 3.3 (p<0.001) when the rescuer had flu-like symptoms. However, the availability of PPE appeared to offset these concerns, with the willingness to perform chest compressions rising back to 91.3% ± 1.8 (p<0.001).

Three-hundred and five (66.7%) participants reported that knowing the victim was vaccinated for COVID-19 increased their willingness to perform CPR during the pandemic. 273 (59.7%) participants felt that education on the risk of COVID-19 transmission would encourage them to perform CPR. When presented with the information that one rescuer may die of COVID-19 out of 10,000 bystander CPR events, while more than 300 lives could be saved [17], willingness dropped from 86.2% ± 2.3 to 69.4% ± 2.4 (p<0.001) for a stranger and from 97.1% ± 1.2 to 74.3% ± 2.4 for a family or friend (p<0.001). We did not detect a difference in the willingness to perform CPR on a stranger during the pandemic depending on whether participants had close relations who had tested positive for COVID-19.

Perceptions of COVID-19 transmission and mitigation strategies

Four hundred and thirty-two (94.5%) participants perceived COVID-19 as airborne or transmittable through contact with the mouth or saliva. One hundred and twenty-nine (28.2%) participants believed it could be transmitted by contact with skin. 409 (89.5%) participants believed handwashing, vaccination, masking, and physical distancing could reduce transmission, while 216 (47.3%) perceived recovery from infection as lowering transmission. Regarding daily precautionary measures, 450 (98.5%) participants reported wearing or carrying a mask when not at home, 345 (75.5%) carried sanitizer, and 43 (9.4%) carried gloves.

## Discussion

Our study highlights the impact of the COVID-19 pandemic on the public's willingness to perform CPR for OHCA. Participants exhibited heightened concern for COVID-19 exposure during more invasive interventions, such as chest compressions and rescue breaths, compared to actions like activating emergency medical services (EMS) or acquiring an AED. This was more pronounced when assisting strangers rather than family members or friends. Interestingly, respondents from Nova Scotia demonstrated a higher willingness to perform CPR, possibly due to lower infection and death rates in the province. This information helps to elucidate factors contributing to reluctance to perform CPR during the pandemic, thereby equipping CPR education organizations with critical insights to design effective interventions to surmount these barriers.

As reflected in our findings, participants' reservations about assisting strangers may be attributed to concerns over their unknown COVID-19 infection or vaccination status and a willingness to accept greater risk when a loved one's life is at stake-ideas that align with prior research [14,18,19]. Respondents from Nova Scotia generally demonstrated a higher willingness to perform various CPR interventions than those from British Columbia and Ontario, which could be explained by regional COVID-19 incidence. At the time of the survey, the reported COVID-19 case rate in Nova Scotia was 709.4 per 100,000 people, significantly lower than in British Columbia (3788.3 per 100,000 people) and Ontario (3955.8 per 100,000 people). Nova Scotia's death rate due to COVID-19 was substantially lower (9.71 per 100,000 people) than in British Columbia (39.7 per 100,000 people) and Ontario (63.5 per 100,000 people) [20]. This lower infection and death rate in Nova Scotia may contribute to a higher willingness to perform CPR due to a perceived lower risk of COVID-19 exposure. Alternatively, provincial variations in CPR education and promotion efforts could be contributing. Further research is needed to delve into these differences, as understanding them could have significant implications for shaping targeted emergency response strategies and public health education campaigns beyond this pandemic.

Participants appeared less inclined to assist senior citizens, age 65 years and older, than adults during the pandemic, possibly due to the increased vulnerability of this population to severe COVID-19 illness [21]. Participants might be apprehensive about contracting COVID-19 from elderly individuals, who are more likely to have frequent contact with healthcare settings and caregivers, potentially heightening their risk of exposure [15,22,23]. The finding aligns with prior research that suggests bystanders are more willing to perform CPR on children and young adults compared to older individuals or socially excluded groups [24]. Interestingly, respondents who had close relations with confirmed COVID-19 cases did not show a significant difference in their willingness to perform CPR on strangers, which could suggest that other factors, such as personal risk perceptions and attitudes towards CPR, may play a more substantial role. Our findings on the use of PPE are consistent with previous research, highlighting the importance of PPE in increasing people's willingness to perform CPR, particularly with strangers whose COVID-19 infection or vaccination status may be unknown [14]. Participants were more concerned about droplet or airborne transmission than through skin contact or fomites, suggesting an understanding of transmission routes influencing their protective measures and readiness to intervene.

We were surprised to learn that awareness of the potential risk of COVID-19 mortality decreased participants' willingness to perform CPR, even though the risk was minimal compared to the possible lives that could be saved through their intervention. Previous research showed that public education campaigns can effectively increase CPR awareness and bystander CPR rates [25,26]. However, few of these campaigns likely include information suggesting risk to the rescuer from intervening. Therefore, public education could emphasize rescuer safety through the appropriate use of PPE and hands-only CPR as a less invasive alternative to save lives while minimizing COVID-19 transmission.

Future research, including longitudinal studies with more diverse samples, is necessary to understand the factors influencing willingness to perform CPR. As the COVID-19 situation evolves, transitioning from a pandemic into a more endemic state, with public health orders lifting, pandemic fatigue rising, and increased availability of COVID-19 treatments and vaccinations, studies could assess how these changes influence public attitudes and willingness towards bystander CPR. Given the increasing number of individuals who have been personally infected, future studies with larger numbers can further explore the impact of personal disease experience on CPR willingness.

Additionally, further investigation is needed to understand the impact of public education campaigns on CPR awareness and bystander CPR rates during pandemics. Previous research showed that individuals confident in CPR are more likely to intervene in a cardiac arrest [24,27]. However, this confidence is not evenly distributed across demographic groups. Older individuals and those from lower socioeconomic backgrounds are less likely to have received CPR training and, consequently, are less confident in their ability [28]. This highlights the need for tailored CPR training to address these groups’ needs.

Our investigation has several constraints. We depended on self-reported data, which may be influenced by social-desirability bias. We did not evaluate CPR proficiency, which may influence willingness. The cohort may not accurately represent the general population as it was a convenience sample recruited through online platforms, and most of our participants are highly educated and vaccinated Canadians with a significant healthcare background. While some might consider our sample's skewed nature a limitation, it could be considered a strength given that individuals from such backgrounds are more likely to provide bystander CPR in the community [24].

A notable limitation of our study is the varying number of respondents for different questions due to incomplete survey responses. While 535 participants began the survey, only 382 completed all questions. Consequently, the sample size for individual analyses fluctuated. We acknowledge that this approach assumes that the data is missing at random. This variability might introduce participation bias and affect the generalizability of our findings. Future studies should consider methods to increase completion rates.

## Conclusions

Our study provides valuable insights into the factors influencing individuals' willingness to perform CPR during the COVID-19 pandemic. Encouraging the implementation of PPE in public areas could enhance the probability of bystander CPR in instances of OHCA. Agencies promoting bystander CPR may consider integrating these findings into education and training programs, ensuring they remain effective during and after the COVID-19 pandemic. Further research should investigate the long-term effects of the pandemic on public willingness to engage in life-saving measures and how this willingness fluctuates with changes in public health guidelines and disease prevalence. Additionally, policies to increase public awareness and training on using PPE during CPR could significantly improve public readiness and safety, ultimately leading to better emergency response outcomes in similar health crises.
